# Preoperative Briganti Nomogram Score and Risk of Prostate Cancer Progression After Robotic Surgery Beyond EAU Risk Categories

**DOI:** 10.3390/medicina60111763

**Published:** 2024-10-27

**Authors:** Antonio Benito Porcaro, Rossella Orlando, Andrea Panunzio, Alessandro Tafuri, Alberto Baielli, Francesco Artoni, Claudio Brancelli, Luca Roggero, Sonia Costantino, Andrea Franceschini, Michele Boldini, Lorenzo Pierangelo Treccani, Francesca Montanaro, Sebastian Gallina, Alberto Bianchi, Emanuele Serafin, Giovanni Mazzucato, Francesco Ditonno, Mariana Finocchiaro, Alessandro Veccia, Riccardo Rizzetto, Matteo Brunelli, Vincenzo De Marco, Salvatore Siracusano, Maria Angela Cerruto, Riccardo Bertolo, Alessandro Antonelli

**Affiliations:** 1 Department of Urology, Azienda Ospedaliera Universitaria Integrata, University of Verona, 37126 Verona, Italy; orlandorossella92@gmail.com (R.O.); alberto.baielli@univr.it (A.B.); francesco.artoni@univr.it (F.A.); brancelli.claudio@gmail.com (C.B.); roggero.luca92@gmail.com (L.R.); soniacostantino1@gmail.com (S.C.); franceschini.dr.andrea.vr@gmail.com (A.F.); bbmichelebb@yahoo.it (M.B.); lp.treccani@gmail.com (L.P.T.); montanarofsca@gmail.com (F.M.); sebastiangallina90@gmail.com (S.G.); alberto.bianchimd@gmail.com (A.B.); serafin.mnl@gmail.com (E.S.); dott.giovannimazzucato@gmail.com (G.M.); francesco.ditonno@icloud.com (F.D.); marianafinocchiaro.4@gmail.com (M.F.); alessandro.veccia@aovr.veneto.it (A.V.); riccardo.rizzetto@aovr.veneto.it (R.R.); vzodemarco@gmail.com (V.D.M.); mariaangela.cerruto@univr.it (M.A.C.); riccardobertolo@hotmail.it (R.B.); alessandro_antonelli@me.com (A.A.); 2 Department of Urology, “Vito Fazzi” Hospital, 73100 Lecce, Italy; panunzioandrea14@gmail.com (A.P.); aletaf@hotmail.it (A.T.); 3 Department of Pathology, Azienda Ospedaliera Universitaria Integrata, University of Verona, 37126 Verona, Italy; matteo.brunelli@univr.it; 4 Department of Life, Health and Environmental Sciences, University of L’Aquila, 67100 L’Aquila, Italy; salvatore.siracusano@univaq.it

**Keywords:** disease progression, minimally invasive surgical procedures, nomogram, prostatectomy, prostate cancer

## Abstract

*Background and Objectives*: We sought to investigate whether the 2012 Briganti nomogram may represent a potential prognostic factor of prostate cancer (PCa) progression after surgical treatment beyond European Association of Urology (EAU) risk categories. *Materials and Methods*: From January 2013 to December 2021, data on PCa patients treated with robot-assisted radical prostatectomy at a single tertiary referral center were extracted. The 2012 version of the Briganti nomogram assessing the risk of pelvic lymph node invasion was used. Here, the nomogram score was evaluated both as a continuous and a categorical variable. The association between variables and disease progression after surgery was evaluated through Cox regression models. *Results*: Overall, 1047 patients were identified. According to the EAU classification system, 297 (28.4%) patients were low-risk, 527 (50.3%) intermediate-risk, and 223 (21.3%) high-risk. The median (interquartile range) 2012 Briganti nomogram score within the investigated population was 3% (2–8%). Median (95% Confidence Interval [CI]) follow-up was 95 (91.9–112.4) months. Disease progression occurred in 237 (22.6%) patients, who were more likely to have an increasing 2012 Briganti nomogram score (Hazard Ratio [HR]: 1.03; 95%CI: 1.01–1.81; *p* = 0.015), independently of unfavorable issues at clinical presentation. Moreover, the nomogram score stratified according to tertiles (<3% vs. 3–8% vs. ≥8%) hold significance beyond EAU risk categories: accordingly, the risk of disease progression increased as the score increased from the first (reference) to the second (HR: 1.50; 95%CI: 1.67–3.72; *p* < 0.001) up to the third (HR: 3.26; 95%CI: 2.26–4.72; *p* < 0.001) tertile. *Conclusions*: Beyond EAU risk categories, the 2012 Briganti nomogram represented an independent predictor of PCa progression after surgery. Likewise, as the nomogram score increased so patients were more likely to experience disease progression. Accordingly, it may allow further stratification of patients within each risk category to modulate appropriate treatment paradigms.

## 1. Introduction

Actually, clinical prostate cancer (PCa) is an epidemic issue of such magnitude in the aging male population that the European Association of Urology (EAU) and the National Comprehensive Cancer Network (NCCN), which are the two main worldwide associations dealing with the subject, are forced to continuously update guidelines in order to address appropriate management and avoid treatment drawbacks, which impair quality of life of patients with relative regret [1,2,3,4]. Accordingly, PCa is classified into prognostic risk categories, which are not equivalent for the two main systems and may include treatment options that vary from monitoring strategies up to active therapies such as radical prostatectomy (RP) eventually associated with extended pelvic lymph node dissection (ePLND), which is most frequently performed by the robot-assisted approach, and radiation therapy [1,2]. Unfortunately, these risk categories are not only equivalent for the two main classification systems, but also heterogenous within each group; as such, further prognostic factors able to stratify subgroups are mandatory in order to avoid overtreatment, and to identify subjects at risk, who are more likely to progress. Although molecular biology is promising, it is still far from clinical routine application, as well as multiparametric resonance imaging (mpMRI) findings are still not always reproducible when comparing multicenter studies [5,6]. In this perspective, simple and reproducible nomograms may help for resolving this task [7]. Accordingly, the 2012 version of the Briganti nomogram predicting the risk of pelvic lymph node invasion (PLNI) at final pathology holds as one of the most effective [8]; it accounts for prostate-specific antigen (PSA), clinical T-stage, primary and secondary biopsy Gleason grade, and percentage of biopsy positive cores (BPCs) [8]. Its role as a prognostic factor has been previously investigated in patients with intermediate-risk PCa [9,10], in whom the nomogram stands out as one of the main tools to decide whether or not to perform ePLND. Specifically, the preoperative nomogram score associated with the risk of disease progression after surgery in both patients exhibiting favorable and unfavorable clinical characteristics, independently of the occurrence of adverse pathology or upgrading issues in the surgical specimen [9,10]. Nevertheless, its application as a prognostic factor beyond standard prognostic risk categories has not yet been studied. Here, we sought to investigate the potential of the 2012 Briganti nomogram as an independent predictor of disease progression after surgery in a large cohort of PCa patients treated at a single tertiary referral center beyond EAU prognostic risk categories.

## 2. Materials and Methods

### 2.1. Patient Population, Data Extraction, and Outcome of Interest

From January 2013 to December 2021, data of 1047 PCa patients treated with robot-assisted RP (RARP) at the Department of Urology of the Integrated University Hospital of Verona were retrospectively evaluated. All patients had available follow-up information, were not under androgen blockade, and did not undergo previous treatments for PCa before surgery. For each patient the following information was available: age (years), body mass index (BMI; kg/m^2^), preoperative physical status assessed by the American Society of Anesthesiologists classification system [11], PSA (ng/mL), prostate volume (PV, mL), BPC (%), tumor stage, assessed according to the Tumor Node Metastasis (TNM, 2017 version, 8th edition) system [12], and grade, assessed by the International Society of Urological Pathology (ISUP) system [13]. Surgery was performed by five skilled surgeons and consisted of RARP eventually associated with ePLND according to guideline recommendations with a template including external iliac, obturator, Cloquet’s and Marcille’s regions [1,2,14,15]. Surgical specimens were evaluated for tumor stage, grade, cancer invasion of surgical margins, and number of counted pelvic lymph nodes, as well as for PLNI by two dedicated pathologists. Accordingly, tumors were staged by the TNM system (2017 version, 8th edition) [12], and graded according to the ISUP system [13]. After discharge, patients were followed up and decisions of further treatments after surgery or in case of disease recurrence or progression were considered in a multidisciplinary setting with the aim to optimize and personalize guidelines recommendations with patient issues [1,2]. The objective of the study was to test whether the 2012 Briganti nomogram [8] may represent an independent predictor of PCa progression after surgery beyond EAU risk categories. Accordingly, the preoperative nomogram score was evaluated both as a continuous and a categorical variable, which was stratified according to tertiles. Disease progression represented the outcome of interest and was defined as the event of biochemical recurrence and/or PSA persistence and/or local recurrence and/or distant metastases.

### 2.2. Statistical Methods

Descriptive statistics included frequencies and proportions for categorical variables. Medians and interquartile range (IQR) were reported for continuously coded variables. Wilcoxon rank sum test and Pearson’s Chi-squared test examined the statistical significance of differences in medians and proportions, respectively. The length of time between surgery and the outcome of interest (PCa progression) or the last follow-up was measured as time to event occurrence. Univariable and multivariable Cox proportional hazards regression models tested the association between the 2012 Briganti nomogram and the risk of PCa progression; main clinical variables were used as confounders. Accordingly, hazard ratios (HRs) and relative 95% confidence intervals (CIs) were evaluated. Eventually, appropriate survival risk curves were generated. IBM-SPSS version 26.0 (IBM Corp., Armonk, NY, USA) was used for all analyses. All tests were two-sided with *p* < 0.05 considered to indicate statistical significance.

## 3. Results

### 3.1. Characteristics of the Patient Population Including All EAU Risk Categories

Demographics and clinical characteristics of the entire patient cohort including 1047 PCa patients treated with RARP eventually associated with ePLND stratified according to EAU prognostic risk categories are presented in Table 1. The intermediate-risk category was the most represented including 50.3% (n = 527) of patients, followed by low-risk (28.4%, n = 297) and high-risk (21.3%, n = 223) categories in that order. Overall, the median (IQR) 2012 Briganti nomogram score was 3% (2–8%); accordingly, it progressively increased along EAU risk categories from 2% (1–3%) in the low-risk to 3% (2–7%) in the intermediate-risk up to 12% (5–22%) in the high-risk category (*p* < 0.001). At final pathology, ISUP grade group 1 through 2–3 up to 4–5 included 527 (50.3%), 294 (28.1%), and 226 (21.6%) cases, respectively. Tumors were not organ-confined in 224 (21.4%) cases including extra-capsular extension and seminal vesicle invasion in 102 (9.7%) and 122 (11.7%) subjects, respectively. Positive surgical margins were detected in 264 (25.2%) cases, while PLNI in 84 out of 666 cases (12.6%) with a median (IQR) number of 25 (19–31) counted lymph nodes.

### 3.2. Prognostic Impact of the 2012 Briganti Nomogram in the Patient Population Including All EAU Risk Categories

Median (95% CI) follow-up was 95 (91.9–98.0) months. Disease progression occurred in 237 (22.6%) patients who were more likely to present with PSA levels higher than 10 ng/mL, palpable cancers with higher percentages of BPCs, and grade groups beyond ISUP 3, as well as with a higher 2012 Briganti nomogram score (7.0%, 2.5–17.0% vs. 3.0%, 2.0–6.0%) compared to their non-progressing counterparts (Table 2). By multivariable Cox regression analyses, the 2012 Briganti nomogram evaluated as a continuous variable achieved the independent predictor status of disease progression, independently of unfavorable issues at clinical presentation (HR: 1.03, 95%CI: 1.01–1.81; *p* = 0.015); accordingly, as the nomogram score increased, so patients were more likely to experience PCa progression (Table 2).

**Figure 1:**
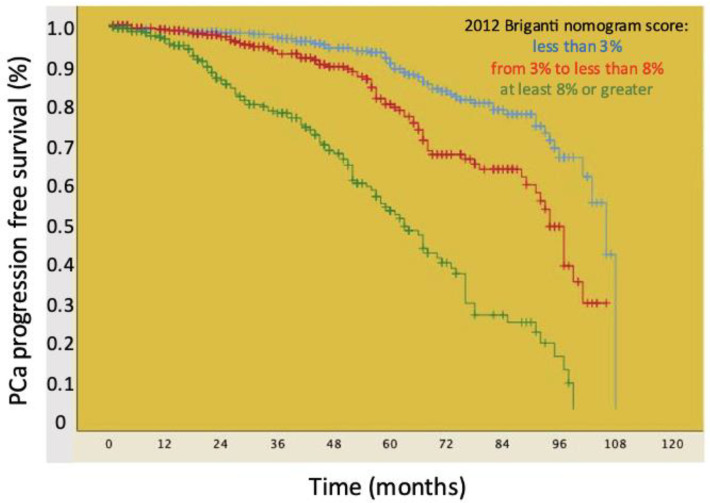
Kaplan–Meier plots depicting PCa progression-free survival in 1047 patients belonging to all European Association of Urology (EAU) prognostic risk categories stratified according to the 2012 Briganti nomogram score distribution based on tertiles: less than 3% vs. from 3% to less than 8% vs. at least 8% or greater.

Figure 1 shows Kaplan–Meier plots depicting PCa progression-free survival of the patient cohort stratified by the 2012 Briganti nomogram score categorized according to tertiles (<3% vs. 3–8% vs. ≥8%). Accordingly, median PCa progression-free survival was higher for patients with a nomogram score <3% (106.0 months, IQR: 99.5–112.4 months) compared to those with a nomogram score 3–8% (94.0 months, IQR: 90.3–97.6 months) and ≥8% (62 months, IQR: 55.8–68.1 months), with the difference being statistically significant (log rank test *p* < 0.001).

The prognostic impact of the 2012 Briganti nomogram categorized according to tertiles beyond EAU risk categories is reported in Table 3; accordingly, the risk of PCa progression increased as the nomogram score ranked from the first (reference) to the second (HR: 1.50, 95%CI: 1.04–2.15; *p* < 0.001) up to the third (HR: 3.26, 95%CI: 2.26–4.72; *p* < 0.001) tertile, independently of EAU risk categories.

## 4. Discussion

The natural history of PCa includes recurrence with progression, which involves approximately 35% of treated cases, till fatal disease, which involves about 16.4% of subjects; likewise, 10-year mortality rates of treated patients may vary from 1.2% through 2.3%, 3.2%, 5.8% up to 13.7% for increasing clinical risk groups according to the Cambridge Prognostic Group classification [1,2,16,17]. As a result, assessing prognostic factors is pivotal for stratifying PCa clinical risk categories; accordingly, although unfavorable tumor grades and PSA dynamics in treated patients predict PCa progression and survival, instead multilevel nomograms including several clinical risk factors have been suggested as the way forward [18,19]. In this perspective, the 2012 Briganti nomogram predicting the risk of PLNI at final pathology in surgically treated patients may also have the potential for predicting disease progression after surgery beyond well-known factors; indeed, it is easy to compute and to reproduce and does not require mpMRI findings, which are not reproducible [8]. We previously tested this hypothesis focusing on patients belonging to the intermediate-risk category, where the decision to perform ePLND is based on a preoperative nomogram score above 5% [8], and demonstrated that it not only predicted PLNI but was also associated with the risk of progression independent of the occurrence of unfavorable features as -non-organ-confined disease, tumor upgrading, or positive surgical margins at final pathology [9,10]. However, it is unknown whether such considerations may also apply to low-risk and high-risk PCa patients. The query has been attacked by the current study which tested the hypothesis in a large population of surgically treated PCa patients belonging to all EAU risk categories.

The study showed that the 2012 Briganti nomogram evaluated either as a continuous (HR: 1.01, 95%CI: 1.01–1.81; *p* = 0.015) or a categorical variable (HR: 1.50, 95%CI: 1.04–2.15; *p* = 0.030 for patients with a nomogram score from 3 to 8% compared to those with a score less than 3%; HR: 3.26, 95%CI: 2.26–4.72; *p* < 0.001 for patients with a nomogram score of at least 8% or greater compared to those with a score less than 3%) represented an independent predictor of PCa progression. Accordingly, as the risk score increased, so patients were more likely to progress independently of the EAU risk category which they belong to at initial diagnosis. These results, tested in a large cohort, represent a novelty, which may impact on managing clinical PCa. Indeed, although molecular biology is the way forward for prognostic stratification of PCa patients, it is still far from daily routine use, and the identification of other simple potential clinical risk factors is advisable for further patient stratification from low- through intermediate- up to high-risk classes involving both EAU and NCCN systems [20,21,22,23,24,25,26,27]. Accordingly, our results have demonstrated that the 2012 Briganti nomogram is further a prognostic factor, which may be managed as a continuous or categorical variable for stratifying prognostic subgroups within each clinical risk category; moreover, it easy to compute and it is not biased by complicated factors included into other nomograms, which are difficult to reproduce. Therefore, our results will turn out useful in daily practice, when counselling patients in order to modulate appropriate treatments; however, confirmatory studies are required. The results of our study also demonstrated that higher nomogram scores positively associated not only with worse clinical risk categories in terms of aggressiveness but also with a more aggressive cancer biology; accordingly, as the nomogram score increased so patients were more likely not only to belong to unfavorable clinical risk categories, but also to experience disease progression. As a theory, these dynamics might be explained by the way the nomogram is patterned; therefore, increasing scores associate with unfavorable cancer biology for interaction and integration at a multidimensional level of each single factor composing the nomogram (PSA, T-stage, primary and secondary biopsy Gleason grade, and BPC), which finally associates with cancers exhibiting genetic instability for high-mutational loads; nevertheless, controlled studies are also needed to test these hypotheses.

Despite the novel findings, this study is not devoid of limitations. First, it is a retrospective and single-center study. Second, mpMRI findings were not evaluated for not being available in all cases; therefore, we did not use the updated version of the nomogram, which specifically accounts for clinical stage and Gleason Grade Group based on MRI data, as well as for maximum diameter of the targeted index lesion at MRI, demonstrating higher accuracy compared to other existing tools [28]. Third, we have defined disease progression as the event of biochemical recurrence and/or local recurrence and/or distant metastases because event numbers prevent us to formally consider these endpoints separately. Fourth, although surgical procedures were performed by several surgeons with different experience, thus reflecting real-world practice at tertiary referral centers, it is possible that it might have affected the pathological evaluation, thus having an impact on disease progression. Finally, median follow-up approximates eight years, which is relatively short compared to 10–15 years that represents the ideal follow-up duration to assess cancer control outcomes, especially when dealing with patients with low- or intermediate-risk disease.

## 5. Conclusions

Beyond EAU prognostic risk categories, the 2012 Briganti nomogram independently predicted PCa progression after surgery. Likewise, as the nomogram score increased so patients were more likely to experience disease progression. Accordingly, it may allow further stratification of patients within each risk category in order to modulate appropriate treatment paradigms.

## Figures and Tables

**Table 1:** Descriptive statistics of demographics and clinical characteristics for the study cohort stratified according to European Association of Urology (EAU) prognostic risk categories.

	EAU Low-Risk PCa n = 297 (28.4%)	EAU Intermediate-Risk PCa n = 527 (50.3%)	EAU High-Risk PCa n = 223 (21.3%)	*p*-Value
Age (years)	65.2 (59.3–69.7)	65.2 (60.1–70.2)	67.7 (61.1–71.2)	**0.036**
BMI (kg/m^2^)	26.1 (23.9–28.4)	25.6 (23.7–27.8)	25.7 (24.2–28.4)	**0.039**
ASA physical status				0.1
ASA I	25 (8.4)	47 (8.9)	16 (7.2)	
ASA II	253 (85.2)	427 (81.0)	178 (79.8)	
ASA III	19 (6.4)	53 (10.1)	29 (13.0)	
PV (mL)	40 (30–50)	38 (30–49)	40 (30–55)	**0.042**
PSA (ng/mL)				**<0.001**
<10	297 (100)	421 (79.9)	126 (56.5)	
10–20	0 (0)	106 (20.1)	47 (21.1)	
>20	0 (0)	0 (0)	50 (22.4)	
BPC (%)	26.6 (14.2–41.6)	28.5 (18.7–47.0)	42.8 (26.6–62.5)	**<0.001**
Clinical T stage				**<0.001**
T1c	225 (75.8)	304 (57.7)	71 (31.8)	
T2	72 (24.2)	223 (42.3)	116 (52.0)	
T3	0 (0)	0 (0)	36 (16.2)	
ISUP grade group				**<0.001**
ISUP 1	297 (100)	35 (6.6)	29 (13.0)	
ISUP 2–3	0 (0)	492 (93.4)	62 (27.8)	
ISUP 4–5	0 (0)	0 (0)	132 (59.2)	
Clinical N stage				**<0.001**
N0	297 (100)	527 (100)	166 (74.4)	
N1	0 (0)	0 (0)	57 (25.6)	
ePLND performed				**<0.001**
No	207 (69.7)	155 (29.4)	19 (8.5)	
Yes	90 (30.3)	372 (70.6)	204 (91.5)	
2012 Briganti nomogram score (%)	2 (1–3)	3 (2–7)	12 (5–22)	**<0.001**

Continuous variables are reported as medians (interquartile ranges) while categorical factors are reported as frequencies (percentages). Abbreviations: PCa, prostate cancer; BMI, body mass index; ASA, American Society of Anesthesiologists; PV, prostate volume; PSA, prostate-specific antigen; BPC, biopsy positive cores; ISUP, International Society of Urological Pathology; ePLND, extended pelvic lymph node dissection. Values in bold indicate statistical significance set at *p* < 0.05.

**Table 2:** Cox regression models testing the association of the 2012 Briganti with prostate cancer (PCa) progression after surgery in 1047 patients belonging to all European Association of Urology (EAU) prognostic risk categories.

	No PCa Progression n = 810 (77.4%)	PCa Progression n = 237 (22.6%)	Univariable Analysis		Multivariable Analysis (*)	
			HR (95% CI)	*p*-Value	HR (95% CI)	*p*-Value
2012 Briganti nomogram score (%)	3.0 (2.0–6.0)	7.0 (2.5–17.0)	1.05 (1.04–1.06)	**<0.001**	1.03 (1.01–1.81)	**0.015**
Age (years)	65 (60–70)	65 (61–70)	1.03 (1.01–1.05)	**0.002**		
BMI (kg/m^2^)	25.8 (23.9–28.1)	25.6 (23.8–28.1)	0.99 (0.95–1.03)	0.5		
PV (mL)	40 (30–50)	39 (30–50)	1.01 (1.00–1.01)	0.2		
PSA (ng/mL)						
<10	691 (85.3)	153 (64.6)	Ref.	-	Ref.	-
10–20	101 (12.5)	52 (21.9)	2.39 (1.74–3.28)	**<0.001**		
>20	18 (2.2)	32 (13.5)	4.23 (2.88–6.20)	**<0.001**	1.44 (1.05–1.97)	**0.023**
BPC (%)	28.5 (16.6–43.7)	42.8 (25.0–64.2)	1.02 (1.02–1.03)	**<0.001**	1.01 (1.00–1.02)	**0.004**
ISUP						
1	308 (38.0)	53 (22.4)	Ref.			
2/3	432 (53.3)	122 (51.5)	2.79 (2.01–3.86)	**<0.001**	2.33 (1.67–3.27)	**<0.001**
4/5	70 (8.7)	62 (26.1)	6.66 (4.59–9.66)	**<0.001**	3.48 (2.26–5.35)	**<0.001**
cT stage						
T1	477 (58.9)	123 (51.9)	Ref.	-	Ref.	-
T2	311 (38.4)	100 (42.2)	2.00 (1.53–2.61)	**<0.001**	1.39 (1.07–1.81)	**0.015**
T3	22 (2.7)	14 (5.9)	4.21 (2.41–7.36)	**<0.001**		
cN stage						
N0	775 (95.7)	215 (90.7)	Ref.	-		
N1	35 (4.3)	22 (9.3)	2.84 (1.82–4.42)	**<0.001**		

Continuous variables are reported as medians (interquartile ranges) while categorical factors are reported as frequencies (percentages). (*), by Wald’s forward method. Abbreviations: HR, Hazard Ratio; CI, Confidence Interval; BMI, body mass index; PV, prostate volume; PSA, prostate-specific antigen; BPC, biopsy positive cores; ISUP, International Society of Urological Pathology. Values in bold indicate statistical significance set at *p* < 0.05.

**Table 3:** Cox regression models testing the independent predictor status of the 2012 Briganti nomogram categorized according to tertiles of prostate cancer (PCa) progression after surgery in 1047 patients belonging to all European Association of Urology (EAU) prognostic risk categories.

	Univariable Analysis		Multivariable Analysis	
	HR (95% CI)	*p*-Value	HR (95% CI)	*p*-Value
**2012 Briganti nomogram score**				
less than 3%	Ref.		Ref	
from 3% to less than 8%	2.02 (1.42–2.87)	**<0.001**	1.50 (1.04–2.15)	**0.030**
at least 8% or greater	5.98 (4.32–8.27)	**<0.001**	3.26 (2.26–4.72)	**<0.001**
**EAU PCa risk categories**				
low-risk	Ref.		Ref	
intermediate-risk	3.20 (2.19–4.69)	**<0.001**	2.51 (1.67–3.72)	**<0.001**
high-risk	8.03 (5.42–11.90)	**<0.001**	4.24 (2.72–6.61)	**<0.001**

Abbreviations: HR, Hazard Ratio; CI, Confidence Interval. Values in bold indicate statistical significance set at *p* < 0.05.

## Data Availability

All data generated or analyzed during this study are included in this article. Further enquires can be directed to the corresponding author.
